# Arterial Hypertension, Metabolic Syndrome and Subclinical Cardiovascular Organ Damage in Patients with Asymptomatic Primary Hyperparathyroidism before and after Parathyroidectomy: Preliminary Results

**DOI:** 10.1155/2012/408295

**Published:** 2012-06-07

**Authors:** Petramala Luigi, Formicuccia Maria Chiara, Zinnamosca Laura, Marinelli Cristiano, Cilenti Giuseppina, Colangelo Luciano, Panzironi Giuseppe, Cerci Sabrina, Sciomer Susanna, Ciardi Antonio, Cavallaro Giuseppe, De Toma Giorgio, Letizia Claudio

**Affiliations:** ^1^Secondary Hypertension Unit, Department of Internal Medicine and Medical Specialties, University of Rome “Sapienza”, Rome, Italy; ^2^Hospital “San Sebastiano” of Frascati, Rome, Italy; ^3^Department of Cardiovascular, Respiratory and Morphological Sciences, University of Rome “Sapienza”, Rome, Italy; ^4^Department of Surgery “P. Valdoni”, University of Rome “Sapienza”, Rome, Italy

## Abstract

*Background*. Primary hyperparathyroidism (PHPT) is associated with high cardiovascular morbidity, and the role of calcium and parathyroid hormone is still controversial. *Objective*. To evaluate the prevalence and outcomes of metabolic syndrome, hypertension, and some cardiovascular alterations in asymptomatic PHPT, and specific changes after successful parathyroidectomy. *Material and Methods*. We examined 30 newly diagnosed PHPT patients (8 males, 22 females; mean age 56 ± 6 yrs), 30 patients with essential hypertension (EH) (9 males, 21 females; mean age 55 ± 4), and 30 normal subjects (NS) (9 males, 21 females: mean age 55 ± 6). All groups underwent evaluation with ambulatory monitoring blood pressure, echocardiography, and color-Doppler artery ultrasonography and were successively revaluated after one year from parathyroidectomy. *Results*. PHPT patients presented a higher prevalence of metabolic syndrome (38%) with respect to EH (28%). Prevalence of hypertension in PHPT was 81%, and 57% presented altered circadian rhythm of blood pressure, with respect to EH (35%) and NS (15%). PHPT showed an important myocardial and vascular remodelling. During follow-up in PHPT patients, we found significant reduction of prevalence of metabolic syndrome, blood pressure, and “non-dipping phenomenon.” *Conclusions*. Cardiovascular and metabolic alterations should be considered as added parameters in evaluation of patients with asymptomatic PHPT.

## 1. Introduction

Primary hyperparathyroidism (PHPT) is an endocrine disease characterized by hypercalcemia due to overproduction of parathyroid hormone (PTH), dependent on single or double adenoma (80–85%), hyperplasia (15–20%), and parathyroid carcinoma (<1%) [1–3].

The clinical presentation of PHPT shows geographic differences, and this disease is accompanied by lack of clinically apparent symptoms, with mild or no signs of complications in a substantial proportion of individuals [4–7]. This chronic disease is associated with high cardiovascular morbidity and mortality [8–11]. The asymptomatic form currently accounts more than 80% of overall PHPT cases.

Some studies have suggested that PHPT is associated with metabolic disorders such as impairment of glucose metabolism, altered lipid profile, hypertension, and structural and functional alterations in cardiovascular system, whereas is uncertain the exact role of calcium and/or PTH in the development of cardiometabolic disorders [12–24].

The aim of the present study was to investigate in a consecutive series of asymptomatic PHPT patients the prevalence of arterial hypertension, metabolic syndrome, and subclinical cardiovascular organ damage, at diagnosis and after successful parathyroidectomy.

## 2. Materials and Methods

Since January 2008 until October 2011, at the Department Unit of Secondary Hypertension, University of Rome “Sapienza”, Italy, we examined 30 consecutive newly diagnosed patients with asymptomatic PHPT (8 males, 22 females; mean age 56 ± 6 yrs).

The PHPT diagnosis was established according to laboratory data characterized by the persistence of high levels of total calcium, ionized calcium, and PTH. All patients underwent neck ultrasonography (US) and/or Tc^99 m^-Sesta MIBI scintiscan imaging. We excluded familial form of PHPT, like multiple endocrine neoplasia (MEN), type 1 or type 2A. The PHPT subjects were defined “asymptomatic” if, without specific lesions of disease, such as kidney stones and bone lesions radiologically evident, were absent.

Office blood pressure (BP) was measured with a standard aneroid manometer with subjects sitting for 5 min, systolic BP (SBP) was taken as the first sound on deflation of the cuff (Korotkoff phase I), and diastolic BP (DBP) was taken as the complete disappearance of Korotkoff sounds (phase V). Hypertension was confirmed by repeated BP measurements of SBP > 140 mmHg and DBP > 90 mmHg [25].

Control groups consist of 30 patients with essential hypertension (EH) (9 males, 21 females; mean age 55 ± 4 yrs) and 30 normal subjects (NS) (9 males, 21 females; mean age 55 ± 6 yrs), who were not affected by hypertension, metabolic syndrome, diabetes mellitus, and cardiovascular diseases. In all subjects, secondary hypertension was excluded on the basis of biochemical, hormonal, and instrumental tests.

No patient had renal insufficiency (serum creatinine level <1.3 mg/dL), diabetes mellitus, thyroid dysfunctions, or major cardiovascular disease. This study was performed according to the Declaration of Helsinki II and approved by the local Ethics Committee. All participant gave informed consent.

### 2.1. Anthropometric Parameters

All patients underwent to assessment of weight (kg), height (cm), body mass index (BMI, expressed in kg/m^2^), waist circumference (WC, cm: measured to a minimum of inspiration to the midpoint of the line joining the last rib and the iliac crest).

### 2.2. Biochemistry

Biochemical variables were determined after an overnight fast by anaerobic sampling, evaluating calcium-phosphorus metabolism (total and ionized calcium, phosphorus, total and ionized magnesium, alkaline phosphatase, parathyroid hormone (PTH), 25 (OH) vitamin D, and alkaline phosphatase (ALP)), renal function (creatinine, blood urea nitrogen, and serum electrolytes), and lipid and glucose metabolisms (total cholesterol, HDL cholesterol, LDL cholesterol, triglycerides, and blood fasting glucose). Patients performed a 24-hour urine collection for determination of urinary excretion of calcium and phosphorous. Ionized calcium was measured with a potentiometric analyzer: the range of this method at pH 7.4 was 1.17–1.33 mmol/L. Intact serum PTH (i-PTH) was measured using a radioimmunoassay method (RIA commercial kits; Diasorin PTH, Still Water, MN, USA).

### 2.3. PHPT Diagnosis and Surgical Treatment

The diagnosis and surgical treatment of asymptomatic PHPT patients have been recently updated by international workshop [26]. The preoperative imaging study (ultrasonography, computed tomography, and MIBI scintigraphy), although not recommended for diagnostic purposes, has been used for localization and mini-invasively treatment, especially in patients who have previously undergone parathyroid or other neck surgery. Intraoperative PTH measurements were used to limit duration of parathyroidectomy. Focused surgery was performed by our specialist surgeon with high experience in this kind of operation [27]. We enrolled PHPT patients with histopathologically confirmed single adenoma and lack of recurrence after followup. We did not evaluate the gland size of parathyroid adenomas.

### 2.4. Bone Mineral Densitometry (BMD)

Bone mineral density (BMD) at lumbar spine (L1–L4) and femoral neck (FN) was obtained in all patients using dual-energy X-ray absorptiometry (DXA) using Hologic QDR-4500 device (Hologic, Inc., Waltham, MA, USA) according to WHO recommendations. The assessment of BMD was expressed as g/cm^2^ and as standard deviation from the mean peak bone mass than healthy adults of the same sex (T-score). The diagnosis of osteoporosis was made in the case of T-score ≤−2.5, and osteopenia if T-score was between −2.5/−1, normal bone mass with superior T-score of −1 [28]. Regarding the precision of BMD evaluation, the coefficient variation was 1% at the lumbar spine and 1.2% at femoral neck side.

### 2.5. Ambulatory Blood Pressure Monitoring (ABPM)

ABPM for 24 h was performed by means of an oscillometric device Space Labs 90207 (Space Labs Medical, Richmond, WA, USA) which was set to measure BP for every 15 min during the day (from 6 : 00 to 22 : 00 hours) and every 30 min during the night (from 22 : 00 to 6 : 00 hours). The definition of “dipper” and “non-dipper” was established where night time SBP and DBP decrease was >10% and <10%, respectively. Subjects without a complete 24 h BP measurement (14 diurnal and 7 nocturnal measurements) repeated the ABPM. The length of hypertension was not evaluated in study groups.

### 2.6. Metabolic Syndrome

Metabolic syndrome was defined according to Adult Treatment Panel III [29] criteria, and its diagnosis required three or more of the following: (1) waist circumference (WC) greater than 102 cm in men and greater than 88 cm in women, (2) triglycerides of 150 mg/dL or higher, (3) high-density lipoprotein (HDL) cholesterol less than 40 mg/dL for men and less than 50 mg/dL for women, (4) fasting glucose of 100 mg/dL or more, and (5) systolic BP of 130 mmHg or more and diastolic BP of 85 mm Hg or more.

### 2.7. Echocardiography

Echocardiography was carried out by same physician operator (SS) in left patient lateral decubitus by a Toshiba echocardiography equipment APLIO with a multifrequency transducer, 2nd harmonic, and TDI implementation, with simultaneous continuous ECG monitoring. Motion mode (M-mode) measurements were recorded through the parasternal view following the recommendations of the American Society of Echocardiography.

Myocardial thickness and left ventricular (LV) diameter at the end of diastole and at the end of systole were measured in M-mode. Left ventricular mass (LVM) was indexed (i) for height and surface area; LVM was determined from the interventricular septum (IVS) and posterior wall thickness (PWLV), diastolic diameters by use of the M-mode formula of Troy. The relative LV wall thickness was calculated as the sum of the interventricular and PW thickness divided by the diastolic diameter. LV values were calculated according to the Teicholz M-mode formula used to calculate the ejection fraction (EF). Aortic (Ao) root diameter was measured at diastole by the leading-edge-to-leading-edge technique at the maximal diameter of the sinuses of Valsalva.

Doppler echocardiographic parameters: early (E) and late (A) mitral and tricuspidal velocities ratio (E/A ratio) isovolumetric relaxation time (IVRT). Right atrium (RA) and left atrium (LA) diameters were also measured. Left ventricular hypertrophy (LVH) was considered present, if the LVMi exceeded 110 g/m^2^ in women and 131 g/m^2^ in man.

### 2.8. Ultrasound Evaluation of the Carotid Arterial Wall

The carotid arteries were examined by ultrasound by same trained operator (MC), and both carotid arteries (common carotid, bifurcation, and internal carotid) were examined in each subject; the mean carotid artery intima-media thickness (IMT) was defined as the overage of 36 IMT readings (common, bifurcation and internal carotid arteries, right and left side, far and near wall, with three sampling points for segment). Plaques were defined as a focal protrusion major of 50% of the surrounding wall. Arterial wall thickening was defined as a mean carotid IMT > 0.90 mm, and it is consistent with the definition of carotid wall thickening proposed in the clinical guidelines for the management of arterial hypertension issued by the European Society of Hypertension and European Society of Cardiology (ESH/ESC). All subjects gave their written consent after explanation of the nature and purpose of the study.

### 2.9. Statistical Analysis

Statistical analysis was performed with Sigmastat program (Jandel Corporation, USA). All data are expressed as mean ± standard deviation (SD). The comparison between groups was performed using the Student *t*-test for variables distributed in the normal way and by the Mann-Whitney test for nonparametric variables. The study of correlations between various parameters was performed using the Spearman test and in case of multiple correlations was performed multivariate analysis using Backward Stepwise Regression method. A *P* < 0.05 was considered statistically significant.

## 3. Results

Results of demographic, biochemical, and cardiovascular parameters are reported in Table 1, We found no differences in all groups for sex and mean age. As expected, we found values of PTH more elevated in patients with PHPT than in those of both control groups, EH and NS (*P* < 0.001, resp.). We observed no statistically significative differences regarding the prevalence of hypovitaminosis D (vitamin D <20 ng/mL) between PHPT patients and control groups (PHPT: 25%; EH: 20%; NS: 19%).

Patients with PHPT and EH showed alterations of fasting blood glucose, total cholesterol, LDL cholesterol, triglycerides, and uric acid serum levels compared to NS (*P* < 0.001, resp.). Moreover, clinical blood pressure (SBP and DBP) values were higher in EH and PHPT patients compared to NS (*P* < 0.001, resp.). However, only in PHPT patients, we revealed higher heart rate (HR) with respect to EH and NS (*P* < 0.001, resp.). In PHPT, hypertension was present in 81% of patients.

**Figure 1:**
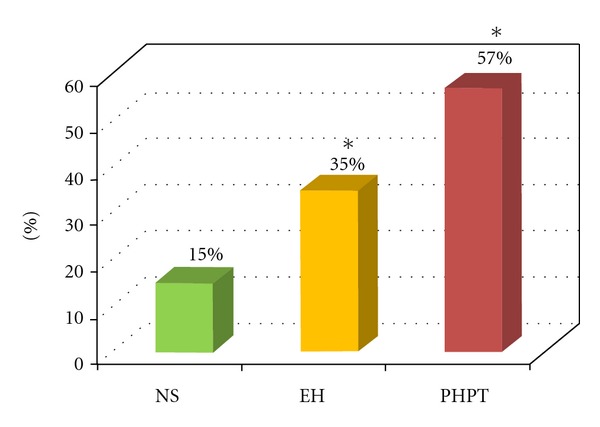
Prevalence of  “non-dipping pattern” in all groups studied. NS: normal subjects; EH: patients with essential hypertension; PHPT: patients with primary hyperparathyroidism. *P = 0.02 versus NS.

**Figure 2:**
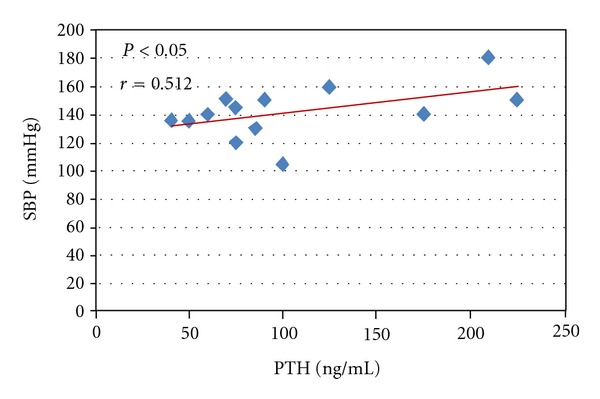
Linear correlation in PHPT patients between PTH levels and systolic blood pressure values (SBP) (*r* = 0.512, *P* < 0.05).

**Figure 3:**
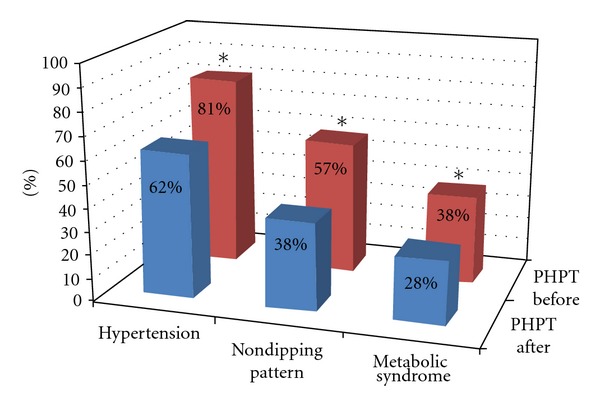
Prevalence of arterial hypertension, “non-dipping pattern,” and metabolic syndrome in PHPT patients before (PHPT before) and after surgery (PHPT after). *P < 0.05.

The ABPM showed significantly higher values of global-SBP (G-SBP), diurnal-SBP (D-SBP) and global DBP (G-DBP) in PHPT and EH patients compared to NS (Table 2). No significant difference were found for ABPM between PHPT and EH patients. At diagnosis, 57% of PHPT patients presented a nocturnal “non-dipping pattern” respect to 35% of EH patients and 15% of NS (Figure 1). Moreover, the correlation study revealed in PHPT patients, a positive correlation between SBP and PTH levels (*r* = 0.512; *P* < 0.05) (Figure 2) The prevalence of metabolic syndrome was present in 38% of PHPT and in 28% of EH patients (Figure 3).

**Figure 4:**
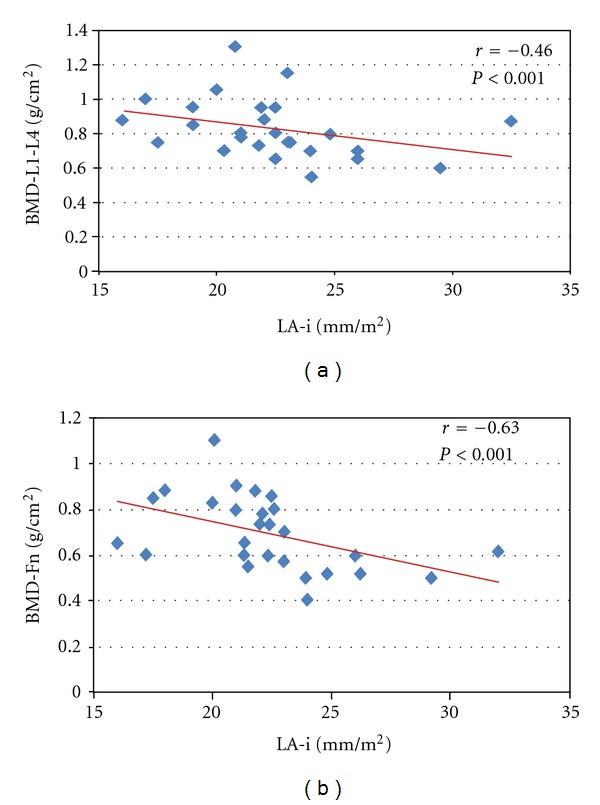
Linear correlation in PHPT patients between left atrium diameter (LAi) and bone mineral density (BMD) at lumbar spine (a) (*r* = −0.46; *P* < 0.01) and femoral neck (b) (*r* = −0.63; *P* < 0.001).

PHPT and EH patients showed an important cardiac remodeling compared to NS, such as significant increase of the IVSi (10.7 ± 0.9 mm and 11 ± 0.9 mm versus 8.8 ± 1.2 mm, resp.; *P* < 0.001), LVMi (182 ± 30.4 mm and 183 ± 63 mm versus 125 ± 26 mm, resp.; *P* < 0.001), and LAi (37.5 ± 3.5 mm and 39 ± 3.8 mm versus 20.8 ± 2.4 mm, resp.; *P* < 0.001) (Table 3). Moreover, 29.3% of PHPT patients showed a calcification of the aorta, and 28.5% had an altered release of the left ventricle (E/A < 1). In PHPT patients, we detected a significant negatively correlations between BMD at lumbar spine (L1–L4) and femoral neck (Fn) with the dimension of LAi (*r* = −0.46, *P* < 0.01; *r* = −0.63, *P* < 0.001, resp.) (Figure 4).

PHPT and EH patients present an IMT of the common carotids significantly greater than NS (0.8 ± 0.3 mm and 0.8 ± 0.1 mm versus 0.6 ± 0.07, resp.; *P* = 0.05). Moreover, 9.7% of PHPT patients had atherosclerotic plaques at arterial carotid bulbs, with positive correlation between 24 h urinary calcium excretion and IMT (*r* = 0.650, *P* < 0.03).

In all PHPT patients, multivariate analysis showed that serum calcium along with the age constitutes an independent factor for cardiac remodelling, such as increase of the IVS and PWLV (Tables 4(a) and 4(b)).

### 3.1. After Parathyroidectomy

All patients underwent mini-invasive parathyroidectomy, with the detection of intraoperative PTH levels. After a mean follow-up of 12 month (range 10–18 months), all PHPT patients had parathyroid adenoma based on pathological examination, showing normalization of serum calcium and PTH levels as normal urinary calcium and phosphorous excretion (Table 5).

There was a significant decrease in clinical SBP and DBP (*P* < 0.001 and *P* < 0.002, resp.) and significant reduction of the SBP-G (126 ± 18 mmHg versus 116 ± 18 mmHg, *P* < 0.001), SBP-D (130 ± 18 mmHg versus 119 ± 15 mmHg, *P* < 0.05), HR-G (81 ± 10 bpm versus 75 ± 10 bpm, *P* < 0.02), and HR-D (85 ± 8.5 mmHg versus 77 ± 6.9 mmHg, *P* < 0.01). Moreover, we also showed significant decrease of “non-dipping pattern” (57% versus 42%; *P* < 0.05).

As well as significant reduction of blood pressure values (decreased prevalence of hypertension from 81% to 62%), we found a significant decrease of mean number of antihypertensive drugs (mean 1.7 versus 1.05; *P* < 0.001), 15% of patients did not take any antihypertensive drug, and 50% patients took less number and dose of antihypertensive drugs (Table 6). Thus, we found an overall reduction of global cardiovascular risk factors in PHPT patients, such as lower prevalence of metabolic syndrome (38% versus 28%).

Regarding echocardiography, in PHPT patients the cardiac parameters did not decrease significantly after 12 months from surgery, except the percentage of E/A ratio (<1) (28.5% versus 12%; *P* < 0.05).

## 4. Discussion

PHPT is a common endocrine disorder, with typical bone involvement represented by a reduced bone mass at skeletal sites rich in cortical tissue [30], and clinical features of hypercalcemia such as fatigue, anorexia, thirst, and polyuria [31]. Asymptomatic subtype (without typical renal and bone involvement) became more frequent over last four decades, probably due to outline determination of serum calcium levels. There is a general agreement that symptomatic PHPT patients should undergo parathyroidectomy [32]. On the other hand, an increased body of evidence showed different metabolic and cardiovascular alterations associated with asymptomatic PHPT, argues in favor of enhanced risk of cardiovascular disorders in this condition [23, 33], especially if these patients did not undergo parathyroidectomy [34, 35]. In particular, these metabolic alterations include insulin resistance [36], diabetes mellitus [37], hyperlipemia [38], arterial hypertension [14, 39, 40], and disorders associated to adiposity, as metabolic syndrome [29]. In our study, we found an higher prevalence of metabolic syndrome (38%) in PHPT patients with respect to EH patients (28%). The prevalence of the metabolic syndrome in our PHPT patients was very higher than reported in a general population with demographic characteristics recruited in Italy [41]. These data confirmed our precedent study [42]. In particular, we showed an high prevalence (29.8%) of metabolic syndrome in PHPT patients, correlated with significant alterations of some fat adipokines, such as leptin and adiponectin. Previous PHPT reports have suggested that common mechanisms are responsible for the development of the metabolic syndrome and some studies examined these cardiometabolic abnormalities in detail [43], showing correlations between PTH and several variables (dyslipidemia, obesity, hypertension, and insulin resistance) observed in the metabolic syndrome, suggesting a role of PHT in development of abnormalities seen in this condition. Indeed, reduction of PTH levels after successful parathyroidectomy is associated to decrease of blood pressure, fasting blood glucose, serum triglycerides, and cholesterol, with reduction of the percentage of the metabolic syndrome (38% versus 28%, resp.).

In our study, we examined the remodelling of cardiac and vascular structure in PHPT patients, compared to EH and NS. In PHPT and EH patients, we showed an alteration of the LVM, IVS, and carotid IMT. Moreover, 29.3% of PHPT patients had a calcification of the aorta and 28.5% had an altered release of the left ventricle (E/A ratio <1). Precedently, some studies have reported a decrease of E/A ratio, which may be a sign of impaired LV diastolic function. In particular, Almquist and coworkers revealed that 83% of the PHPT patients have an E/A ratio less than 1 [44]. Dalberg et al. reported lower E/A ratio among PHPT patients compared to controls, but the blood pressure was significantly higher in these patients [45], while some studies have reported no differences between PHPT patients and controls with E/A ratio [46].

The cause and detailed characteristics of cardiovascular derangement in PHPT have been incompletely clarified. The high prevalence of LVH in PHPT patients has been attributed, in part, to effects of PTH and/or hypercalcemia [3]. In fact, it has been shown that PTH has a direct effect on cardiomyocytes through the activation of the kinase C-protein [47] with an increase of cellular protein mass. Moreover, other authors suggest that the increase in cardiac mass could be secondary to a higher incidence of arterial hypertension in PHPT patients [35]; in patients recruited in the present study, there is a large share of hypertensive patients. PHPT is associated with increased risk of arterial hypertension, and recent investigations have reported high blood pressure in 56% and 80% of PHPT patients [48–50]. In particular, Broulik et al. showed high prevalence (68.9%) of arterial hypertension in PHPT patients, with significant reduction of clinical mean SBP and SDP values after successful parathyroidectomy [51]. Several studies have demonstrated that hypertensive individuals with a “non-dipping” blood pressure pattern show an increased frequency of target organ damage, such as LVH and carotid IMT [52]. In the present study, we revealed that 57% of PHPT patients presented “non-dipping” blood pressure pattern with respect to 35% of EH patients and 15% of NS. In PHPT patients, this phenomenon was reduced after parathyroidectomy (38%). During ABPM registration, in PHPT group, we found a significantly higher heart rate during 24 hours and daytime registration with respect to EH and NS, significantly reduced after surgical treatment of PHPT.

The increased risk of death observed in PHPT patients could be referred to higher bioelectrical risk due to enhanced sympathetic activity; in previous work, we investigated sympathovagal balance (heart rate variability and QT parameters) in asymptomatic PHPT patients compared to NS, showing in PHPT enhanced sympathetic tone (shorter QTc interval, higher QTc dispersion, and lack of physiological adaptation of QT length to R-R interval), restored after parathyroidectomy. Moreover, Hysing et al. have suggested that slight increase of ionized calcium was associated to higher heart rate probably due to autonomic nervous system hyperactivity [53]. These findings confirmed increased bioelectrical risk to life-threatening arrhythmias in PHPT patients and bioelectrical instability induced by hypercalcemia, gradually reduced after parathyroidectomy [16, 17].

Serum calcium could be implicated in the pathogenesis of arterial hypertension in PHPT [54]. Some authors suggested that PHPT increases calcium influx into the cell and, consequently, its vasoconstriction. Previously, we reported a positive correlation between serum-ionized calcium and blood pressure in PHPT patients, suggesting that the increase of serum-ionized calcium may be an independent factor of peripheral resistance elevation [14]. In the present study, we revealed, in PHPT patients, a positive correlation between SBP and PTH values before parathyroidectomy (Figure 2). These results are similar to data reported by Farahnak et al. [55] that showed, in the PHPT group, SBP values were correlated to the levels of PTH and calcium, significantly higher compared to controls, and decreased after successful parathyroidectomy. However, the LVH in PHPT may be related to hypercalcemia secondary autonomous overproduction of PTH. This hypothesis is supported, in part, by our results. In fact, in PHPT patients at the multiple regression analysis, the IVS and PWLV are correlated only with serum-ionized calcium and age.

In this study, carotid IMT is increased with respect to NS, and positively correlated with the urinary calcium excretion. These data extended and confirmed other results reported in the literature [56].

Finally, in our study, another important result found is the relation between BMD, specific target of PHPT, and some echocardiographic parameters, typical feature of organ damage due to cardiovascular disease and metabolic disorders. In fact, we showed negative correlations between BMD values (at lumbar and femoral sites) and LA diameter. Left atrial function has a large contribution in left ventricular diastolic function. Increased atrial response to early-stage left ventricular filling impairment is characterized by augmented reservoir and pump functions, according to a Starling mechanism, which becomes hardly effective at end-stage ventricular dysfunction when the limits of the atrial preload reserve are reached. When left ventricular filling pressure was increased, the E/A ratio increases, indicating a filling shift towards early diastole. Significant changes in left atrium on diastolic function that we have observed in our PHPT patients may contribute to failing left ventricular filling and itself may undergo failure. In our patients, we found a significant reduction of E/A ratio after successful parathyroidectomy.

Bone densitometry is an important component in the evaluation of the bone metabolism in PHPT patients, because it has more sensitivity than conventional radiograms. In PHPT, skeletal compromise is demonstrated by reduced BMD values (osteopenia or osteoporosis), evident especially at the cortical bone (i.e., femoral). There is a cross-sectional association between BMD loss and aortic calcification, carotid plaques, IMT, and coronary calcification [57, 58]. In our study, 29.3% of PHPT patients showed a calcification of aorta. The mineral within calcified atherosclerosis plaques is hydroxyapatite, the same mineral as in bone and matrix vesicles. Specific factors and protein crucial to bone function are also present in atherosclerotic lesions. The vascular calcification of the aorta was showed greater in patients with the amount of bone loss, and the progression of calcification is associated with increased bone loss in women during menopause [59–61]. Probably, the mobilization of calcium from the skeleton in PHPT may be responsible for its deposition in the atherosclerotic plaque and several mechanisms may explain this association, especially regarding the role of PTH. The increment of PTH could be responsible for a decrease in cortical bone and for an increment in cardiovascular risk too.

## 5. Conclusions

Significant findings in our asymptomatic PHPT patients are as follow: (1) about 38% of patients presented a metabolic syndrome as defined by ATP III criteria; (2) high proportion of PHPT patients was hypertensive, with altered circadian rhythm of blood pressure; (3) asymptomatic PHPT is associated with cardiovascular manifestations, such as LVH and increased carotid intima-media thickness, subclinical predictors of cardiac and cerebrovascular events; (4) relation between BMD (at lumbar and femoral site) and some echocardiographic parameters (LA) is evident; (5) parathyroidectomy significantly reduces blood pressure associated to “non-dipping phenomenon” and metabolic syndrome.

These metabolic and cardiovascular alterations should be considered as added parameters in therapeutic evaluation of patients with asymptomatic PHPT. Other studies are necessary in order to fully establish the indications towards parathyroidectomy in asymptomatic PHPT patients.

## Figures and Tables

**Table 1:** Demographic, biochemical, calcium-phosphorus mineral metabolism, and bone mineral density in studied groups.

	Age (yrs)	Sex (M/F)	BMI (kg/m²)	WC (cm)	SBP (mmHg)	DBP (mmHg)	HR (bpm)
PHPT (n.30)	54 ± 12	8 M/22 F	27.4 ± 4.4	95.7 ± 11.8	144 ± 10.2*	90.4 ± 10.3*	75 ± 6.4*
EH (n.30)	55 ± 5	9 M/21 F	27.1 ± 2.3	90 ± 3.5	135.3 ± 5*	84.4 ± 5*	71 ± 8
NS (n.30)	55 ± 6	9 M/21 F	26.1 ± 2.19	85.7 ± 2.19	129.3 ± 4	78.4 ± 4	69 ± 9
*P*	ns	ns	ns	ns	*<0.001 versus NS	*<0.001 versus NS	*<0.001 versus EH-NS

	Creatinine (mg/dL)	Fasting blood glucose (mg/dL)	CT (mg/dL)	LDL-C (mg/dL)	HDL-C (mg/dL)	TG (mg/dL)	Uric acid (mg/dL)

PHPT (n.30)	0.96 ± 0.4	92.6 ± 7.5*	227.4 ± 26.2*	146 ± 21.3*	54 ± 6.2	135 ± 21.7*	6.9 ± 2.6*
EH (n.30)	1.02 ± 0.03	102.5 ± 14.5*	217 ± 41.3*	133.7 ± 36.4*	55.3 ± 13.9	140.7 ± 33.5*	5.07 ± 2.1*
NS (n.30)	0.94 ± 0.3	83.9 ± 4	192 ± 17.5	116.8 ± 18.5	57.4 ± 7.4	94.5 ± 16.1	3.6 ± 1
*P*	ns	*0.001 versus NS	*0.001 versus NS	*0.001 versus NS	ns	*0.001 versus NS	*0.001 versus NS

	Calcium (mg/dL)	Ca^++^ (mmol/L)	Phosphorous (mg/dL)	Mg^++^ (mmol/L)	Ca Ur (mg/24 h)	PTH (pg/mL)	ALP (UI/L)

PHPT (n.30)	11.2 ± 1.2*	1.51 ± 0.2*	2.73 ± 0.8*	0.45 ± 0.06*	352 ± 177*	122 ± 47.7*	154 ± 74.6*
EH (n.30)	9.8 ± 0.2	1.22 ± 0.03	2.95 ± 0.3	0.53 ± 0.03	170 ± 18.3	32 ± 5.3	112 ± 57
NS (n.30)	9.4 ± 0.3	1.21 ± 0.02	3.43 ± 0.36	0.52 ± 0.04	155 ± 17.3	29 ± 2.39	100 ± 52
*P*	*<0.001 versus EH-NS	*<0.001 versus EH-NS	*0.006 versus EH-NS	*0.006 versus EH-NS	*<0.001 versus EH-NS	*<0.001 versus EH-NS	*0.016 versus EH-NS

	BMD L1-L4 (gr/cm^2^)	BMD Fn (gr/cm^2^)					

PHPT (n.30)	0.870 ± 0.15*	0.750 ± 0.14*					
EH (n.30)	0.980 ± 0.1	0.83 ± 0.11					
NS (n.30)	1 ± 0.09	0.82 ± 0.08					
*P*	*<0.001 versus EH-NS	*<0.001 versus EH-NS					

BMI: body mass index; WC: waist circumference; SBP: systolic blood pressure; DBP: diastolic blood pressure; HR: heart rate; CT: total cholesterol; LDL-C: low-density cholesterol; HDL-C: high-density cholesterol; TG: triglycerides; ns: not significative; Ca^++^: serum-ionized calcium; Mg^++^: serum-ionized magnesium; Ca Ur: calcium urinary excretion in 24 hours; PTH: serum parathyroid hormone; ALP: serum phosphatase alkaline; BMD: bone mineral density at lumbar spine (L1–L4) and femoral neck (Fn).

**Table 2:** Ambulatory blood pressure monitoring (ABPM) during 24 hours in studied groups.

	SBP-G (mmHg)	DBP-G (mmHg)	HR-G (bpm)	SBP-D (mmHg)	DBP-D (mmHg)	HR-D (bpm)	SBP-N (mmHg)	DBP-N (mmHg)	HR-N (bpm)
PHPT (n.30)	126 ± 18*	73 ± 21*	81 ± 10*	130 ± 18*	82 ± 10	85 ± 8.5*	117 ± 18	67±17	72 ± 8.5
EH (n.30)	131 ± 19*	82 ± 11*	75 ± 10	137 ± 15*	86 ± 11	78 ± 11	123 ± 18	74 ± 12	69 ± 10
NS (n.30)	116 ± 4	74 ± 5	75 ± 6	119 ± 4	77 ± 5	77 ± 7	109 ± 7	69 ± 6	69 ± 5
*P*	*<0.001 versus NS	*<0.001 versus NS	*<0.002 versus EH-NS	*<0.001 versus NS	ns	*<0.004 versus EH-NS	ns	ns	ns

SBP-G: global systolic blood pressure; DBP-G: global diastolic blood pressure; HR-G: global heart rate; SBP-D: diurnal systolic blood pressure; DBP-D: diurnal diastolic blood pressure; HR-D: diurnal heart rate; SBP-N: nocturnal systolic blood pressure; DBP-N: nocturnal diastolic blood pressure; HR-N: nocturnal heart rate.

**Table 3:** Echocardiographic parameters in studied groups.

	IVSi (mm/m²)	PWLVi (mm/m²)	LV-DTDi (mm/m²)	LV-DTSi (mm/m²)	LAi (mm/m²)	EF (%)	LVMi (mm/m²)	IMT (mm)
PHPT (n.30)	10.7 ± 0.9*	10.4 ± 1.4*	46 ± 5.9*	30 ± 2.4*	37.5 ± 3.5*	59 ± 3.2	182 ± 30.4*	0.8 ± 0.3*
EH (n.30)	11 ± 0.9*	10.7 ± 1.1*	48 ± 3.9*	32 ± 3.4*	39 ± 3.8*	59 ± 3.3	183 ± 63*	0.8 ± 0.1*
NS (n.30)	8.8 ± 1.2	8.9 ± 1.2	29.3 ± 1.9	18.3 ± 1.9	20.8 ± 2.4	58 ± 2.5	125 ± 26	0.6 ± 0.07
*P *A-B versus C	*<0.001 versus NS	*0.01 versus NS	*<0.001 versus NS	*<0.001 versus NS	*<0.001 versus NS	ns	*<0.001 versus NS	*<0.05 versus NS

IVSi: interventricle septum; PWLVi: posterior wall; LV-DTDi: telediastolic diameter of left ventricle; LV-DTSi: telesystolic diameter of left ventricle; LAi: left atrium; EF: ejection fraction; LVMi: mass of left ventricle indexed; IMT: intima-media thickness.

**Table:** (a) Multiple regression analysis between interventricular septum (IVS) and metabolic variables in PHPT patients.

Variables	Coefficient	*P* value
Age (years)	0.0390	0.043
BMI (kg/m²)	−0.0186	0.699
Calcium ionized (mmol/L)	7.288	0.012
PTH (pg/mL)	0.00357	0.453
Fasting blood glucose (mg/dL)	0.0657	0.056
Total cholesterol (mg/dL)	0.0188	0.889
LDL-cholesterol (mg/dL)	−0.0242	0.121
Uric acid (mg/dL)	−0.0732	0.630
Systolic blood pressure (mmHg)	0.00527	0.217
Diastolic blood pressure (mmHg)	−0.0000196	0.368

**Table:** (b) Multiple regression analysis between posterior wall of the left ventricle (PWLV-i) and metabolic variables in PHPT patients.

Variables	Coefficient	*P* value
Age (years)	0.0282	0.050
BMI (kg/m²)	0.0421	0.346
Calcium ionized (mmol/L)	5.183	0.048
PTH (pg/mL)	0.00598	0.374
Fasting blood glucose (mg/dL)	0.0455	0.211
Total cholesterol (mg/dL)	0.0197	0.241
LDL-cholesterol (mg/dL)	−0.0249	0.657
Uric acid (mg/dL)	0.0734	0.635
Systolic blood pressure (mmHg)	0.00620	0.149
Diastolic blood pressure (mmHg)	−0.0176	0.589

**Table 5:** Demographic, biochemical, ABPM, echocardiographic, IMT parameters in PHPT patients before (PHPT pre) and after (PHPT post) surgery.

	WC (cm)	SBP (mmHg)	DBP (mmHg)	HR (bpm)	Glycaemia (mg/dL)	CT (mg/dL)	LDL-C (mg/dL)	HDL-C (mg/dL)	TG (mg/dL)
PHPT pre (n.30)	95.7 ± 12	144 ± 19.2	90.4 ± 10.3	75 ± 9.4	92.6 ± 7.5	227 ± 26	146.1 ± 42	54 ± 6.2	135 ± 22
PHPT post (n.30)	95 ± 10.3	117 ± 19.2	83 ± 14.3	65 ± 8.1	87.9 ± 10.2	209 ± 25	132 ± 23	57 ± 12	118 ± 35
*P*	ns	0.001	0.002	ns	ns	ns	ns	ns	ns

	Calcium (mg/dL)	Ca^++^ (mmol/L)	Phosphorous (mg/dL)	Magnesium (mg/dL)	Mg^++^ (mmol/L)	Ca Ur (mg/24 h)	P Ur (mg/24 h)	PTH (pg/mL)	ALP (UI/L)

PHPT pre (n.30)	11.2 ± 1.2	1.51 ± 0.2	2.73 ± 0.8	1.94 ± 0.21	0.45 ± 0.06	353 ± 177	829 ± 340	122 ± 47	154 ± 75
PHPT post (n.30)	8.9 ± 2.1	1.2 ± 0.08	3.3 ± 0.76	1.77 ± 0.33	0.51 ± 0.07	126 ± 96	578 ± 277	50.9 ± 22	106 ± 80
*P*	<0.001	<0.001	<0.001	ns	0.001	<0.001	0.003	<0.001	ns

	SBP-G (mmHg)	DBP-G (mmHg)	HR-G (bpm)	SBP-D (mmHg)	DBP-D (mmHg)	HR-D (bpm)	SBP-N (mmHg)	DBP-N (mmHg)	HR-N (bpm)

PHPT pre (n.30)	126 ± 18	73 ± 21	81 ± 10	130 ± 18	82 ± 10	85 ± 8.5	117 ± 18	67 ± 17	72 ± 8
PHPT post (n.30)	116 ± 18	73 ± 15	75 ± 10	119 ± 15	77 ± 10	77 ± 6.9	109 ± 16	67 ± 16	70 ± 5
*P*	0.001	ns	0.002	0.001	ns	<0.01	ns	ns	ns

	IVSi (mm/m²)	PWLVi (mm/m²)	LV-DTDi (mm/m²)	LV-DTSi (mm/m²)	LAi (mm/m²)	EF (%)	LVMi (mm/m²)	IMT (mm)	

PHPT pre (n.30)	11 ± 0.9	10 ± 1.4	46 ± 5.9	30 ± 2.4	37.5 ± 3.5	59 ± 3.2	182 ± 30	0.8 ± 0.3	
PHPT post (n.30)	11 ± 1.2	10 ± 1.1	46 ± 2.2	29.6 ± 3.2	37.5 ± 2.9	56.8 ± 5	176 ± 32	0.7 ± 0.2	
*P*	ns	ns	ns	ns	ns	ns	ns	ns	

WC: waist circumference; SBP: systolic blood pressure; DBP: diastolic blood pressure; HR: heart rate; CT: total cholesterol; LDL-C: low-density cholesterol; HDL-C: high-density cholesterol; TG: triglycerides; ns: not significative; Ca^++^: serum-ionized calcium; Mg^++^: serum-ionized magnesium; Ca Ur: calcium urinary excretion in 24 hours; P Ur: phosphorous urinary excretion in 24 hours; PTH: serum parathyroid hormone; ALP: serum phosphatase alkaline; SBP-G: global systolic blood pressure; DBP-G: global diastolic blood pressure; HR-G: global heart rate; SBP-D: diurnal systolic blood pressure; DBP-D: diurnal diastolic blood pressure; HR-D: diurnal heart rate; SBP-N: nocturnal systolic blood pressure; DBP-N: nocturnal diastolic blood pressure; HR-N: nocturnal heart rate; IVSi: interventricle septum; PWLVi: posterior wall; LV-DTDi: telediastolic diameter of left ventricle; LV-DTSi: telesystolic diameter of left ventricle; LAi: left atrium; EF: ejection fraction; LVMi: mass of left ventricle indexed; IMT: intima-media thickness.

**Table 6:** Antihypertensive drugs taken by PHPT patients at baseline (before) and after 12 months from surgery.

	AT1 receptor antagonists	Ace inhibitor	*α*-Blocker	Diuretic	Calcium antagonist	*β*-Blocker
Before	42%	28.6%	9.5%	33%	19%	4.7%
After	33%	4.7%	9.5%	19%	14%	4.7%
	−21%	−83.5%	—	−42%	−26%	—
